# Comparison of the clinical efficacy of ultrasound-guided GON blockade using low and high concentrations of bupivacaine in chronic migraine

**DOI:** 10.55730/1300-0144.5782

**Published:** 2023-11-18

**Authors:** Ümit Murat PARPUCU, Onur KÜÇÜK, Fatih SAĞ, Suna Akın TAKMAZ, Barış KIRAN, İsmail Eren DURMUŞ, Yılmaz KARADUMAN, Semih AYDEMİR

**Affiliations:** 1 Department of Anesthesiology and Reanimation, Gülhane Faculty of Health Sciences, University of Health Sciences, Ankara, Turkiye; 2 Department of Anesthesiology and Reanimation, Ankara Atatürk Sanatoryum Training and Research Hospital, University of Health Sciences, Ankara, Turkiye; 3 Clinic of Anesthesiology and Reanimation, Tavşanli Assoc. Doc. Mustafa Kalemli State Hospital, Kütahya, Turkiye; 4 Department of Anesthesiology and Reanimation, Ankara Training and Research Hospital, University of Health Sciences, Ankara, Turkiye; 5 Clinic of Neurology, Çan State Hospital, Çanakkale, Turkiye; 6 Department of Anesthesiology and Reanimation, Antalya Training and Research Hospital, University of Health Sciences, Antalya, Turkiye

**Keywords:** Chronic migraine, greater occipital nerve block, local anesthetic concentration, peripheral nerve block

## Abstract

**Background/aim:**

In this study, it was aimed to retrospectively compare the effect of greater occipital nerve (GON) block performed with ultrasonography using low (0.3%) and high (0.5%) concentrations of bupivacaine on pain scores and patient satisfaction in chronic migraine (CM).

**Materials and methods:**

The mean number of days with pain, the mean duration of pain in the attacks, and the highest numerical rating scale (NRS) scores recorded in the 1 month preblock and 1 and 3 months postblock of 80 patients (40 for Group 1, 0.3% bupivacaine; 40 for Group 2, 0.5% bupivacaine) who underwent ultrasonography-guided GON block were recorded from the patient file data. According to the protocol applied by our clinic, GON block was applied to each patient 6 times with the same procedures, in total.

**Results:**

While there was a statistically significant difference between the groups in terms of the number of days with pain and the maximum NRS score in the 1-month preblock evaluation (p = 0.01, p < 0.001), at 3 months postblock, no statistical difference was observed in terms of the number of days with pain, duration of pain, or NRS score (p = 0.961, p = 0.108, and p = 0.567). In the intragroup evaluations, at 3 months postblock, the number of days with pain decreased from 17.5 days to 7 days in Group 1 and from 24.0 days to 8.0 days in Group 2. The duration of pain and maximum NRS values were statistically significantly decreased in the intragroup evaluation in both groups pre and postblock.

**Conclusion:**

Complications arising from the procedure and the local anesthetic used are essential points to consider in applying GON block. In CM treatment using GON block application, a similar effect to the standard local anesthetic application (0.5%) can be achieved by administering local anesthetic at a lower dose (0.3%).

## 1. Introduction

Migraine is a chronic, neurovascular brain disorder with significant effects on patients and society [1]. It affects around 15% of the global population and is typically characterized by recurring, severe headaches, nausea, vomiting, extreme sensitivity to light and sound, and other variable physical, mental, and psychological symptoms, often resulting in high levels of disability. Chronic migraine (CM) is a primary headache disorder that can be diagnosed when patients experience fifteen or more headache days per month for more than 3 months, with at least 8 of those days exhibiting migraine features. The condition is more common in women than in men and has the highest prevalence between the ages of 18 and 50 [2]. CM negatively affects daily life, reduces the quality of life, and causes loss of workforce [3].

Etiology and risk factors can be counted as inadequate treatment of acute migraine pain, high-dose drug intake in treating acute migraine attacks, obesity, depression, and a stressful lifestyle [4]. In the treatment, pharmacological approaches such as antiepileptics, antihypertensives and antidepressants, botulinum toxin-A injection, neuromodulation, blockade of the greater occipital nerve (GON), supraorbital and vagal nerve stimulation and surgical approaches can be applied [4–6]. The GON, small occipital nerve, supraorbital nerve, supratrochlear nerve, auriculotemporal nerve, and sphenopalatine ganglion blockade can be applied in patient groups where conventional and standard treatment approaches are insufficient [4–6].

GON blockade is one of the interventional methods with proven effectiveness in migraine and similar primary headaches and is the most common nerve block in CM. A metaanalysis of 2864 studies conducted in 2022 indicated that GON block therapy with local anesthetics can decrease the frequency and intensity of headaches in the treatment of CM [7]. The GON originates from the second and third cervical nerves and is the primary sensory nerve of the occipital region. The mechanism by which GON blockade treats CM involves the convergence of central connections with the spinal nucleus of the trigeminal nerve, specifically the nucleus caudalis [7]. Local anesthetics reversibly inhibit nerve conduction by blocking voltage-gated calcium channels. This reduction in afferent stimuli from GON-innervated regions inhibits the excitation of convergent neurons in the C2 dorsal horn [7,8]. GON blockade has also been effective in cervicogenic headaches, occipital neuralgia, cluster headache, and migraine [6]. When studies in the literature were examined, it was observed that GON blockade with various local anesthetics is effective in reducing the frequency, severity, and duration of migraine attacks [9]. In GON blockade, lidocaine, prilocaine, and bupivacaine can be applied with local anesthetic agents and adjuvants such as corticosteroids [10]. Systemic and depot corticosteroids have been studied as a potential adjuvant therapy due to their local antiinflammatory effects [8]. Both drugs alleviate pain by intervening in the pathophysiology of migraines [8,11]. However, studies have shown that adding corticosteroids to local anesthetics does not provide additional benefits [7]. In most of the studies, bupivacaine was applied at a concentration of 0.5%.

Thus, this study aimed to compare the effectiveness of a low (0.3%) and high (0.5%) concentration of bupivacaine in GON blockade patients with CM.

## 2. Materials and methods

### 2.1. Study population and study design

This research was designed as a retrospective, single-center observational efficacy study. The study began after approval was obtained by the Health Sciences University Ankara Training and Research Hospital ethics committee, dated 29/09/2021 and numbered E-93471371-14.01.02. All of the procedures performed complied with the ethical standards of the institutional and/or national research committee and the 1964 Helsinki Declaration (as revised in 2013) and its subsequent amendments or comparable ethical standards.

Patients aged between 18 and 65 years who were followed-up with a diagnosis of CM according to the International Headache Classification-3 (ICHD-3) and who underwent ultrasonography-guided GON blockade for prophylactic treatment were included in the study. Patients over 65 years of age, under 18 years of age, with a history of primary headache other than CM, with a history of secondary headaches, who had previously undergone occipital nerve blockade or stimulation, who had used any prophylactic treatment for headache in the last 30 days preceding treatment/follow-up (patients taking medication for headache), who received analgesics for any reason at any point during the 3-month treatment/follow-up period, who had undergone surgery from the occipital region patients with a history of malignancy, who had a history of local anesthetic allergy, who were pregnant and/or lactating, who had chronic kidney failure or liver disease, who were receiving botulinum toxin type A (BoNT-A) therapy, who were using anticoagulant or antiaggregant therapy, who had a history of bleeding diathesis, who had major psychiatric disease (major depression, etc.), who had Arnold Chiari history, who had neuromuscular dysfunction, who were using agents that affect neuromuscular functions such as aminoglycoside or antibiotics such as aminoglycoside, and who had mental retardation were excluded from the study. Hence, the data of 80 patients were included in the study.

### 2.2. Clinical protocol

CM is defined as a headache that lasts for more than 3 months and occurs for more than 15 days per month, with migrainous characteristics present for at least 8 days per month [2]. Diagnostic criteria for CM, according to the ICHD-3 criteria, are outlined in 4 main headings (A, B, C, D) [2], which are A: “headache on ≥15 days/month for >3 months, and fulfilling criteria for B and C”; B: “occurring in a patient who has had at least 5 attacks fulfilling criteria for migraine with aura or migraine without aura”; C: “on 28 days/month for >3 months, fulfilling any of the following: criteria C and D for migraine without aura, criteria B and C for migraine with aura, believed by the patient to be migraine at onset and relieved by a triptan or ergot derivatives”; and D: “not better accounted for by another ICHD-3 diagnosis”. CM diagnosis at our clinic is confirmed through consultation with the neurology department.

In our clinic, patients who had been diagnosed with CM according to the ICHD-3 criteria by the neurology clinic received ultrasound-guided GON block for prophylactic treatment. Randomly given was 1.5 mL of 0.3% bupivacaine + 1.5 mL of saline to odd-numbered patients, and 1.5 mL of 0.5% bupivacaine + 1.5 mL of saline to even-numbered patients during the day, and all of the patients were followed-up routinely. GON block was applied to each patient in the same way, 6 times in total, every week in the first month and once a month in the second and third months. During the 1 month preblock and at 3 months postblock, the patients’ pain frequency, intensity, number of painful days, and numerical rating scale (NRS) values were recorded. At 3 months postblock, the patients were evaluated using a 3-point Likert-type question scale, which was defined as dissatisfied (1 point), undecided (2 points), and satisfied (3 points).

### 2.3. Working groups and block implementation

Patients who received 1.5 mL of 0.3% bupivacaine + 1.5 mL of saline were categorized as Group 1 (n = 40), while those who received 1.5 mL of 0.5% bupivacaine + 1.5 mL of saline were categorized as Group 2 (n = 40) for the GON block application.

Intravenous vascular access was provided to the patients. Pulse oximetry and electrocardiography monitoring were performed. GON block was applied with the classical approach. The external occipital protuberance was palpated, and the intervention area was cleaned using an antiseptic solution. Each patient sat in a chair and flexed their head on their arms placed on the examination table during injection to avoid trauma to syncope. Bupivacaine injection was performed 2-cm lateral and 2-cm caudal to the occipital prominence with a 22-G spinal needle under ultrasound guidance. Local pressure was applied for 2–3 min to spread the solution and prevent bleeding. After the application, a hypoesthesia examination was performed on the GON trace. After the interventional procedure, the patients were followed-up in the ward for observation for 30 min.

### 2.4. Compared data

Age, sex, pain frequency, pain intensity, number of days with pain, NRS values, and patient satisfaction values at 1 month preblock were compared with those at 3 months postblock.

Measurement of patient satisfaction is important following regional analgesia administration. Researchers in the literature have utilized visual analog scales (VAS) or Likert-type scales ranging from 3 to 5 points to measure this phenomenon [12]. Herein, patient satisfaction was evaluated in the context of treatment following GON block using a 3-point Likert patient satisfaction scale.

### 2.5. Statistical analysis

IBM SPSS Statistics for Windows 20.0 (IBM Corp., Armonk, NY, USA) was used for statistical analysis of the data. Descriptive statistics were expressed as the mean ± standard deviation (SD) or median (interquartile range) according to the normal distribution of the continuous variables, and the descriptive statistics of the categorical data were expressed in the form of numbers and percentages. The normality distribution of the data was evaluated using the Kolmogorov–Smirnov test and the homogeneity of the variances was evaluated using the Levene test, whereas the Mann–Whitney U test was used to compare the nonnormal numerical data and Student’ t test was used to compare the normal data. Either a chi-squared test or Fisher’s exact test were used to evaluate the categorical data. p < 0.05 was considered statistically significant.

## 3. Results

After analysis of the data, it was found that the mean age for those treated with 0.3% bupivacaine was 38.1 (±10.3) years in Group 1, of whom 82.5% were female, while in Group 2, the mean age was 42.5 (±13.5) years, with 80% being female. Statistical analysis determined no significant difference between the groups in terms of age or sex (p = 0.143, p = 0.775, respectively) (Table 1).

Preblock, the median number of painful days in the 1-month period was 17.5 days (14.0–24.5) for Group 1 and 24 days (16.0–30.0) for Group 2. There was a statistically significant difference between the groups (p = 0.01), and Group 2 had a statistically significantly higher number of painful days. The maximum preblock NRS value was 8 (7–9) in Group 1 and 6 (6–7) in Group 2, with a statistically significant difference (p < 0.001). The highest preblock NRS value was significantly greater in Group 1. There was no statistical significance in pain duration preblock between the groups (p = 0.07). Detailed preblock values of the groups are presented in Table 2.

The results indicated that there was no statistically significant distinction in the number of painful days between the groups at 3 months postblock (p = 0.961). Pain durations were comparable between the groups at 3 months postblock (p = 0.108). Additionally, there was no statistically significant difference between the groups in the maximum NRS values measured during the 3-month postblock follow-up (p = 0.567) (Table 3). At 3 months postblock, the data showed no significant difference between the groups according to the satisfaction scale results obtained via the 3-point Likert questionnaire (p = 0.646). In Group 1, 92.5% (n = 37) of the patients expressed satisfaction with the treatment, compared to 95% (n = 38) in Group 2.

In the current investigation, significant reductions in the number of painful days, pain duration, and maximum NRS values were found in both the low-dose (0.3%) and high-dose (0.5%) bupivacaine groups compared to the 3 month postblock follow-up values. Although there was a significant difference between the groups preblock, there was no statistically significant difference detected in the treatment response after postblock. During the intragroup evaluations, there was a significant decrease in the number of painful days for both groups at 3 months postblock (p < 0.001). In Group 1, the number of painful days decreased from 17.5 days (14.0–24.5) to 7 days (6.0–9.5), and in Group 2, the number of painful days decreased from 24.0 days (16.0–30.0) to 8.0 days (5.0–12.0). Additionally, the pain durations and maximum NRS values also significantly decreased in both groups preblock and at 3 months postblock (p < 0.001 and p < 0.001, respectively). In the evaluation within each group (refer to Tables 4 and 5), there were statistically significant treatment responses at 3 months postblock for both groups.

Finally, no complications were observed during or after the block procedure.

## 4. Discussion

GON blockade is a proven intervention in treating CM. According to the results of the current study, nerve blockade with 0.3% and 0.5% bupivacaine was found to be effective in treating CM. GON blockade is accepted as an interventional approach that has been shown to be effective in treating migraine. However, the efficacy of the injection attempt in different headache clinics remains uncertain. Some studies recorded the definitions of GON blockade and migraine pain as suboccipital nerve blockade and different headache clinics. Studies in this area must be controlled and meticulously planned [10].

CM significantly impairs patients’ quality of life and socioeconomic functioning. The main modifiable risk factors for CM are overuse of acute migraine medication, ineffective acute treatment, obesity, depression, and stressful life events [13]. The use of oral migraine medication is particularly challenging. Overconsumption of migraine medication is also linked to CM [2]. Using analgesics for more than 15 days per month or triptans for more than 10 days per month is classified as acute medication overuse headache and is the most crucial cause of CM [2]. Moreover, regular use of migraine medications leads to an increased frequency of headaches, and this accelerates the progression of migraine [14–16]. Therefore, discontinuing acute drug overuse not only offers significant headache relief but also enhances the efficacy of prophylactic migraine treatments [16]. The processes involved in GON block therapy make it an effective treatment for CM. GON blockade operates by altering nociceptive pathways in the brain and regulating common noxious inhibitors [17,18]. The trigeminocervical complex is connected to the nucleus salivatorius through the raphe nucleus, locus coeruleus, and hypothalamus, as is widely acknowledged. Painful stimuli originating from cranial structures transmit via the trigeminal nerve and superior cervical nerve to the trigeminocervical complex, and eventually upper centers [9,19]. In humans, there exists a significant functional relationship between the sensory occipital segments and the trigeminal nociceptive system. As a result, GON blockade is a viable treatment choice for CM patients, effectively shielding them from severe issues [19]. In the current study, the efficacy of GON block application was investigated with different local anesthetic doses in the treatment of CM. No other medication was given to the patients before, during the treatment, or in the follow-up period. With this approach, it was aimed to maximize the effectiveness of our treatment.

In a study by Gül et al. [9] in which bupivacaine and GON blockade were compared with a placebo in CM patients, the VAS score was significantly decreased in the bupivacaine group compared to placebo. In the present study, a significant improvement in VAS values and pain intensity was observed in the saline group for 1 month postblock compared to preblock. In a study conducted by Inan et al. [18] with 84 patients, GON was blocked with bupivacaine once a week for a total of 4 times in one group, while the other group was blocked with saline fluid. In the bupivacaine group, pain intensity, pain duration, and VAS score were statistically significantly decreased compared to the placebo group. In the current study, similar improvements were observed in the pain clinic during the 3-month postblock follow-up.

In the treatment of CM, the local anesthetic agent used in the GON blockade approach is 0.5% bupivacaine [20]. In the study herein, no significant difference was found between the groups in terms of CM clinic, and it was shown that the groups that were blocked with 0.3% and 0.5% bupivacaine exhibited effective treatment of CM pain. The NRS value, pain clinic, and patient satisfaction were statistically similar in both groups.

Complications related to GON blockade in the treatment of migraine can be listed as local anesthesia toxicity, bradycardia, and syncope. Vertigo, alopecia, cutaneous atrophy, facial oedema, and sleep disturbances can be seen due to the processing and steroids used as adjuvant [21, 22]. No adjuvant was used in the current study. Furthermore, no complications related to GON block application were documented.

GON blockade is an invasive procedure conducted proximal to the occipital artery. Although arterial hematoma is rare, it is considered a predictable complication [23]. In the current research, vascular access was established for emergency intervention in all of the patients and the block was performed under ultrasound guidance. To mitigate against the various possible complications, it is our suggestion that the patient have vascular access during the GON block procedure, follow-up, and the block is performed under ultrasound guidance.

There were limitations to this study. First, the pharmacological agents utilized by the patients prior to and during treatment/follow-up were not compared, and the body mass index (BMI) data of the patients were unavailable. Patients who received pharmacological treatment within 1 month before and during treatment/follow-up were excluded from the study to minimize the effects of pharmacological agents on GON block therapy. BMI data for the patients could not be compared due to unavailability within our registry system. This may have impacted the effectiveness of the GON block. This study was retrospective and, as such, patients with characteristics that could have impacted the effectiveness of GON block treatment were excluded. This could have introduced selection bias, but it may have led to greater generalizability of the effectiveness of local anesthetic dosage in GON block application for CM pain, which was our main objective of the study. Furthermore, due to the retrospective nature of the study, the physicians who executed the block were the same individuals who assessed the data. Nevertheless, the high standardization and effectiveness of the GON block can be attributed to the expertise and interest in algology of the physicians who executed it. It is worth noting that the successful implementation of GON block is highly dependent on treatment methodology, as demonstrated in the pertinent literature [24]. The treatment duration for GON block in CM therapy is 3 months. Patients must adhere to the treatment regimen regularly. The data of patients who failed to complete the 3-month treatment or who discontinued treatment were not analyzed. Therefore, it is essential to note that patient compliance is critical in the application of GON block therapy for CM. To address the uncertain variables concerning the quantity and potency of local anesthetic agents administered in GON blockade procedures, there is a necessity for double-blind randomized prospective studies comprising a larger participant pool.

## 5. Conclusion

Blockade of the GON with bupivacaine was found to be effective in the treatment of patients with CM pain, and there was no difference between the high (0.5%) and low (0.3%) concentrations of bupivacaine in terms of the NRS scores or pain clinic at 3 months postblock. In the treatment of CM pain, it is our belief that GON blockade with low-concentration bupivacaine under ultrasound guidance will be as effective as high-concentration bupivacaine.

## Figures and Tables

**Table 1:** Comparison of the age and sex characteristics between the groups.

	Group 1 (0.3%) n = 40	Group 2 (0.5%) n = 40	p-value
Age, mean ± SD	38.1 ± 10.3	43.6 ± 13.9	0.143
Sex, male/female, n (%)	33 (82.5%)/7 (17.5%)	32 (80.0%)/8 (20.0%)	0.775

Student’s t test, p < 0.05 was considered significant. Pearson chi-squared test, p < 0.05 was considered significant.

**Table 2:** Comparison of the criteria before the block application between the groups.

Preblock	Group 1 (0.3%) n = 40	Group 2 (0.5%) n = 40	p-value
Number of painful days, median (Q1–Q3)	17.5 (14.0–24.5)	24.0 (16.0–30.0)	0.012
Pain duration, hour, median (Q1–Q3)	11.5 (8.0–15.0)	8 (8.0–12.0)	0.066
Maximum NRS value*, median (Q1–Q3)	8 (7–9)	6 (6–7)	<0.001

* Maximum Numerical Pain Severity Scale value stated by the patients before the block.

Mann–Whitney U test, p < 0.05 was considered significant. NRS: Numerical Rating Scale.

**Table 3:** Results of the pain examination evaluation of the patients at 3 months postblock.

Postblock (at 3 months)	Group 1 (0.3%) n = 40	Group 2 (0.5%) n = 40	p-value
Number of painful days, median (Q1–Q3)	7 (6.0–9.5)	8 (5.0–12.0)	0.961
Pain duration, hour, median (Q1–Q3)	6 (5.0–8.0)	6 (4.0–8.0)	0.108
Maximum NRS value *, median (Q1–Q3)	5 (5.0–5.5)	5 (4.0–6.0)	0.567
Patient Satisfaction**, n (%)			0.646
- 2	3 (7.5%)	3 (5.0%)	
- 3	37 (92.5%)	38 (95.0%)	

* Maximum Numerical Pain Severity Scale value stated by the patients during the 3-month follow-up period.

** At the end of 3 months, the satisfaction result of the 3-point Likert-type question scale about the treatment process of the patients.

Mann–Whitney U test, p < 0.05 was considered significant. Pearson chi-squared test, p < 0.05 was considered significant.

**Table 4:** Evaluation at 3 months pre and postblock in Group 1.

Group 1 (0.3%), n = 40	Preblock	Postblock	p-value
Number of painful days, median (Q1–Q3)	17.5 (14.0–24.5)	7.0 (6.0–9.5)	<0.001
Pain duration, hour, median (Q1–Q3)	11.5 (8.0–15.0)	6.0 (5.0–8.0)	<0.001
Maximum NRS value, median, median (Q1–Q3)	8.0 (7.0–9.0)	5.0 (5.0–5.5)	<0.001

Mann–Whitney U test, p < 0.05 was considered significant.

**Table 5:** Evaluation at 3 months pre and postblock in Group 2.

Group 2 (0.5%), n = 40	Preblock	Postblock	p-value
Number of painful days, median (Q1–Q3)	24.0 (16.0–30.0)	8.0 (5.0–12.0)	<0.001
Pain duration, hour, median (Q1–Q3)	8.0 (8.0–12.0)	6.0 (4.0–8.0)	<0.001
Maximum NRS value, median (Q1–Q3)	6.0 (6.0–7.0)	5.0 (4.0–6.0)	<0.001

Mann-Whitney U test, p < 0.05 was considered significant.
